# The 15th ISCB Student Wikipedia Competition: past, present and future perspectives

**DOI:** 10.1093/bioadv/vbag103

**Published:** 2026-05-20

**Authors:** Alastair M Kilpatrick, Audra Anjum, Dan DeBlasio, Pradeep Eranti, Aarón Gallego Crespo, Megha Hegde, Tülay Karakulak, Nicolas C Näpflin, Nelly Sélem-Mojica, Lonnie R Welch, Farzana Rahman

**Affiliations:** Centre for Regenerative Medicine, Institute for Regeneration and Repair, The University of Edinburgh, Edinburgh, EH8 8AQ, United Kingdom; Office of Instructional Design, Ohio University, Athens, OH 45701, United States; Ray and Stephanie Lane Computational Biology Department, Carnegie Mellon University, Pittsburgh, PA 15213, United States; Université Paris Cité, Paris, F-75006, France; Department of Hematology and Oncology, University Medical Center of the Johannes Gutenberg University, Mainz, 55131, Germany; School of Computing and Mathematics, Faculty of Engineering, Computing, and Environment, Kingston University London, London, KT12EE, United Kingdom; Department of Molecular Life Sciences and Swiss Institute of Bioinformatics, University of Zurich, Zurich, CH-8057, Switzerland; Department of Molecular Life Sciences and Swiss Institute of Bioinformatics, University of Zurich, Zurich, CH-8057, Switzerland; Centro de Ciencias Matemáticas, Universidad Nacional Autónoma de México, Morelia, 58089, México; Biomedical Engineering, School of Electrical Engineering and Computer Science, Ohio University, Morelia OH 45701-2979, United States; School of Computing and Mathematics, Faculty of Engineering, Computing, and Environment, Kingston University London, London, KT12EE, United Kingdom

## Abstract

The International Society for Computational Biology (ISCB) Student Wikipedia Competition, now in its 15th edition, has become a cornerstone initiative for promoting open educational resources in computational biology. Launched in 2012, the competition has engaged hundreds of students and postdoctoral researchers worldwide in editing and improving Wikipedia articles, guided by the society’s bioinformatics core competencies. This paper traces the competition’s evolution from its early focus on English-only entries to the inclusion of non-English tracks and collaborative group contributions. It also examines the present format of the competition, effort related to integration of Wikipedia editing into educational curricula, and the persistent challenge of sustaining long-term volunteer engagement. Looking ahead, the article explores the potential influence of artificial intelligence and large language models on scientific content creation, alongside the importance of diversity and regional outreach in addressing global knowledge gaps. Taken together, these perspectives underscore the competition’s educational value, its contribution to open science, and its enduring role in shaping the next generation of computational biologists.

## Introduction

Founded in 1997, the International Society for Computational Biology (ISCB) is the largest professional society in the fields of bioinformatics and computational biology (Rost 2013). As of mid-2025, ISCB has more than 4100 members worldwide, including researchers, technicians, practitioners, suppliers, and students (https://www.iscb.org/iscb-membership). An important tenet of ISCB’s strategic mission is to advance scholarship and training in computational biology through disseminating high-quality information and actively facilitating educational resources and community activities. Several society activities further this strategic mission, ranging from international and regional scientific conferences to an online training and workshop programme, ISCBtv (Capella-Gutierrez *et al.* 2022).

The ISCB Student Council has organized an annual symposium for the last 20 years to foster the development of the next generation of computational biologists (Rahman *et al.* 2015, Hassan *et al.* 2018, Al Sium *et al.* 2024). Another student-focused educational initiative is the ISCB Student Wikipedia Competition (Bateman *et al.* 2013). The competition, open to all students and postdocs, aims to improve Wikipedia’s coverage of any topic relating to the ISCB’s Bioinformatics Core Competencies (Welch *et al.* 2014, 2016, Mulder *et al.* 2018, Brooksbank *et al.* 2024). Given that Wikipedia is a vital educational resource for communicating science to the public, the competition has played a vital role in integrating the contributions of computational biology students and postdocs towards enhancing the quality and visibility of their field in this resource.

September 2025 marks the start of the 15th ISCB Student Wikipedia Competition. To celebrate this milestone, here the ISCB Wikipedia Committee reflects on the history of the competition, with the input of previous competition organisers and facilitators, competition winners, and educators who have incorporated the competition into their classroom activities. We discuss the current status of the competition and suggest possible directions for future editions of the competition and the committee more widely.

## Past

Alex Bateman (Senior Team Leader at EMBL-EBI) was one of the founders of the ISCB Wikipedia Competition. In an interview with the ISCB Wikipedia Committee, Alex reflected on his early experience of Wikipedia:

I don’t think it was for science that I first discovered Wikipedia, it was maybe around 2007 and it started appearing as the first hit in Google searches. Then Wikipedia started coming up in searches for science topics and sometimes the articles were quite reasonable, but sometimes they weren’t that great. So that was sort of the beginning of thinking about (encouraging scientists to write Wikipedia articles).

The origins of the competition came from a proposal at an ISCB Board of Directors meeting. Alex recalled:

I proposed (the competition) and I remember Burkhard Rost, who was ISCB president at the time, was extraordinarily supportive of the idea. The whole board really got behind it, and they gave us prize money for it, which was pretty generous. So a lot of people helped put it together, including Geoff Macintyre, who was involved with the Student Council.

The first ISCB Wikipedia Competition officially started on 9 September 2012, coinciding with the 11th European Conference on Computational Biology. As part of the conference, Alex and Daniel Mietchen held a workshop on Editing Wikipedia for Scientists (Schwede and Iber 2012). The competition closed four months later, on 10th January 2013. Competition entries were reviewed by volunteers from the ISCB Student Council, who compiled a shortlist of the six best articles; these were judged by a panel consisting of computational biology postdocs and researchers, including Burkhard Rost.

The competition closed four months later, on 10th January 2013. Competition entries were reviewed by volunteers from the ISCB Student Council, who compiled a shortlist of the six best articles; these were judged by a panel consisting of computational biology postdocs and researchers, including Burkhard Rost.

The third competition, starting in 2014, introduced two new competition tracks. For the first time, entries were encouraged in languages other than English, with prizes available for the top two non-English articles. A third competition track for “multimedia” entries was also introduced, to encourage the creation of illustrations or otherwise suitably licenced media files for upload to Wikimedia Commons. Again, prizes were available for the top two multimedia entries. Participation in the non-English track in this edition was limited. One attendee of an Australian Computational Biology and Bioinformatics Student Society (COMBINE) Wikipedia editathon claimed an article in German Wikipedia but ultimately made no edits. Two other articles in Swedish Wikipedia were edited by a non-student Wikipedia administrator. While prizes were not awarded in either new track due to low entry numbers, entries in languages other than English were encouraged in subsequent competitions.

We speculate that the higher barrier to entry for non-English speakers was a major factor in the low participation numbers in the non-English track. For instance, we acknowledge that less available guidance on Wikipedia editing in non-English languages may have deterred potential entrants. The 2015 competition merged the English and non-English tracks. Again, due to low participation, the multimedia track was replaced with a Wikidata track. This step was taken to encourage the creation of knowledge base items relevant to computational biology (Kilpatrick 2016). Although prizes were awarded in this category for this edition of the competition, low entry numbers in subsequent competitions (again in retrospect, likely due to higher technical barriers to entry) led to the removal of this track in 2018.

Alex Bateman stepped down as ISCB Wikipedia Committee chair in February 2017, and the role was subsequently taken over by Alastair Kilpatrick (University of California, San Diego, now The University of Edinburgh) and Lonnie Welch (Stuckey Professor of Electrical Engineering and Computer Science, Ohio University), sharing co-chair responsibilities. Alastair described the changeover:

Alex had an increase in his other commitments and didn’t want to be a bottleneck on the competition, so he suggested I organize the competition with Lonnie’s assistance. Lonnie and I had both been involved in judging competition entries previously, so it was a natural choice.

Alastair and Lonnie were joined by an almost completely refreshed judging panel, with Ann Meyer (OICR, Canada) joining as co-chair of the judging committee. Joining the judging panel was Farzana Rahman (University of South Wales, now Kingston University), who would join the ISCB Wikipedia Committee as a third co-chair in 2020. Alongside the personnel changes, the timing of the competition was also changed in 2018, settling into a September to May editing period to match the academic calendar. In 2022, as part of a restructuring and expansion of the ISCB Wikipedia Committee, Tülay Karakulak (University of Zurich) and Nelly Sélem-Mojica (UNAM, Mexico) joined as competition co-chairs. Tülay remarked:

I love organizing meaningful initiatives for students, and encouraging students to engage with science communication and contribute to open knowledge, so joining the Committee as a competition co-chair felt like a natural step for me.

**Figure 1:**
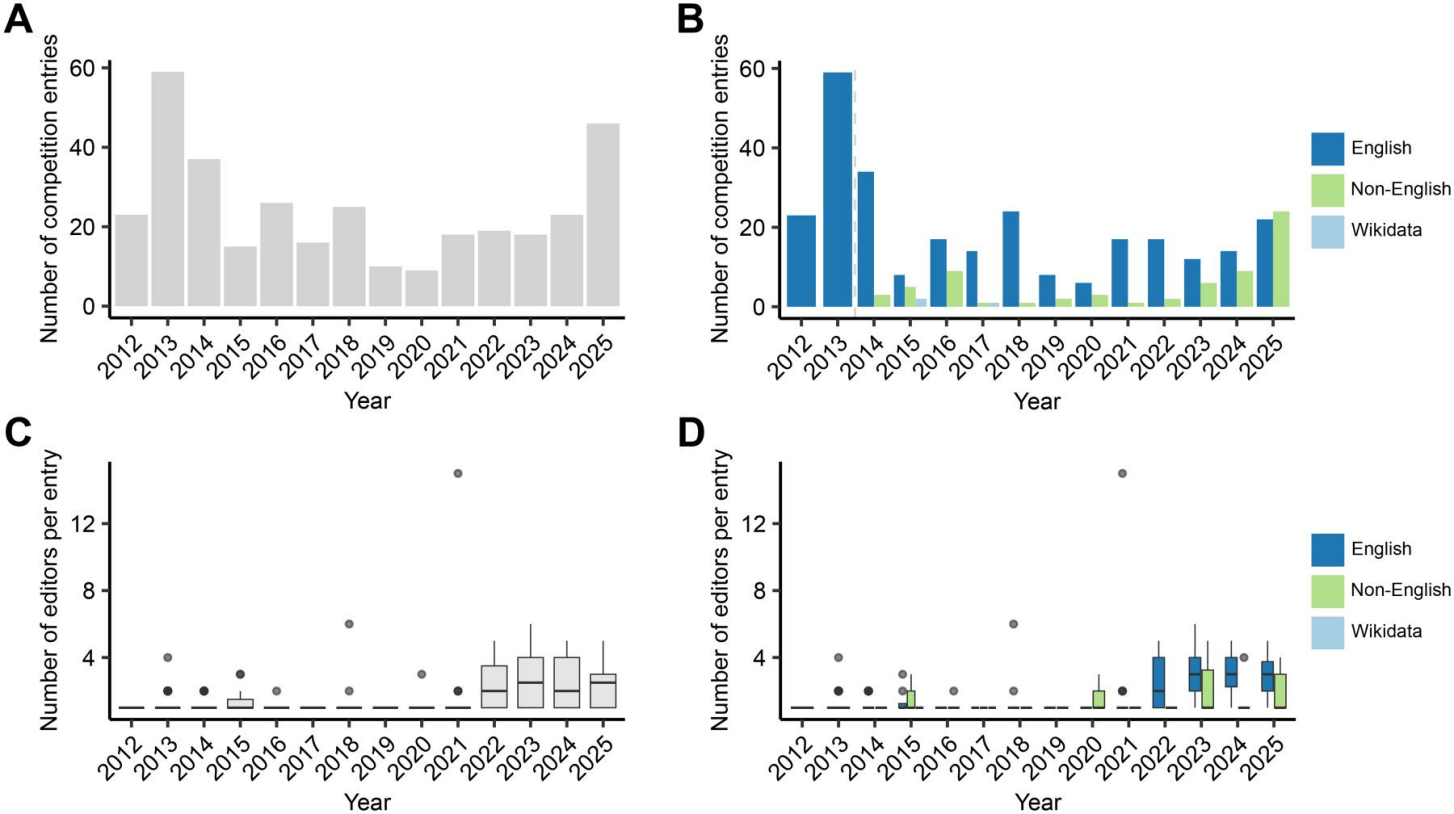
(A) Barplot of number of entries per competition; line represents year end ISCB membership. (B) Barplot of number of entries per competition, split by track. (C) Boxplots illustrating the number of editors per entry, for each competition. (D) Boxplots illustrating the number of editors per entry for each competition, split by track.

Since its launch in 2012, the competition has attracted 344 entries from 433 unique Wikipedia users, editing 302 unique articles (Fig. 1A). The recent rise in competition entries is reflective of an increasing trend in ISCB membership since a drop in 2020–21, potentially driven by the COVID-19 pandemic. However, we note that students and postdocs consistently make up less than half of the overall membership, and that ISCB membership is not necessarily reflective of the overall computational biology community. Since 2021, entries in languages other than English have shown an upward trend (Fig. 1B). During the same period, we have also noticed a growing number of team entries to the competition (Fig. 1C), especially in the English language (Fig. 1D).

## Present

The present competition format is consistent with those in previous years. Entrants select an article (or articles) to edit and make their edits within the competition period. Following the competition period, the difference in article quality between the start and end dates is reviewed. As in previous years, the competition offers a global track and a non-English track. English Wikipedia articles are eligible for awards in the global track, while articles in non-English Wikipedias are eligible for awards in both tracks. However, each article can win in only one track.

In many ways, the present challenges for the competition remain the same as in previous years. Despite recent growth in participant numbers and increasing participation by bioinformatics and computational biology educators as competition judges and coordinators, one perennial challenge is improving global participation numbers to the levels we believe could be achievable and sustainable long-term. Relatedly, a valuable aspect of the competition for learners is the affordance of opportunities for authentic, situated learning that ultimately incentivizes advances in scholarship and participation in the fields of bioinformatics and computational biology. We recognise that integrating these activities into a traditional, or formal, curriculum can be daunting, for both educators and students. Dan DeBlasio, Assistant Teaching Professor (Ray and Stephanie Lane Computational Biology Department at Carnegie Mellon University), has incorporated Wikipedia editing in his classes. Dan reflected:

For students, not only are you writing something that will be read by people you don’t know, but depending on your knowledge level, adding to existing articles on a topic you’re slightly familiar with may be non-trivial. As an educator, the main challenge is timing. Introducing editing assignments too early into a semester risks not giving enough time to provide background to students before they select topics and begin editing. Too late, and not enough time is provided to make substantive changes.

Since joining the ISCB Wikipedia Committee, Lonnie has also been a strong advocate of using Wikipedia editing in formal educational settings, with students working both in editing and in peer-reviewing roles. Lonnie recounted:

We have found in-class editathon sessions to be a valuable educational activity, both for empowering students with the knowledge of Wikipedia editing tools and rules, and to perform peer-review of articles. Working with an instructional designer has been invaluable, resulting in Wikipedia editing course modules that enhance the depth of student learning and in rubric-based assessments. The ISCB Wikipedia Competition has provided an exciting opportunity for both undergraduate and graduate student editors in Bioinformatics and Data Science classes. Based on our years of experience with classroom Wikipedia editing, we have incorporated our knowledge into helpful resources for other educators (Kilpatrick *et al.* 2020, Sélem-Mojica *et al.* 2025).

Despite declining numbers of Wikipedia editors overall, we have experienced growth in our initiative. In 2019, the former WikiProject Computational Biology [a group of editors with expertise in computational biology and bioinformatics (O’Neill *et al.* 2017)] was merged with other related WikiProjects as “taskforces” of a parent WikiProject Molecular Biology, due to falling numbers of active WikiProject editors. However, the number of active WikiProject Molecular Biology editors has not significantly increased since then, and input into the competition organisation from the Wikipedia community beyond the ISCB Wikipedia committee has been minimal in recent years. Investment and focus by the ISCB would be beneficial in these areas. For example, ISCB COSIs could form subcommittees that take ownership of pages relevant to their areas of focus (Kilpatrick *et al.* 2022).

In 2023, Tülay and Nelly reinstated a dedicated “international” track for non-English entries to the competition, which remains to date. The ISCB Wikipedia Committee have also forged links with the ISCB’s Equity, Diversity and Inclusion (EDI) Committee, with a view to narrowing the “knowledge gap” in computational biology coverage between English and non-English Wikipedias through encouraging competition entries in languages other than English. Recent editions of the competition have highlighted the increasing impact of the dedicated international track. However, as Tülay described, this brings its own challenges:

The non-English competition track is a great way to improve diversity in computational biology on Wikipedia, by welcoming articles in any language. But with that comes the challenge of finding reviewers who can adequately assess the quality of those non-English entries. Since the competition is completely volunteer-based, we rely on these reviewers, who play a significant role in sustaining this initiative.

We also acknowledge that the task of reviewing non-English competition entries is an addition to the workload of our reviewing volunteers, who mainly come from communities traditionally underrepresented in computational biology. We have made efforts to increase our pool of reviewers to reduce the burden on these volunteers.

**Figure 2:**
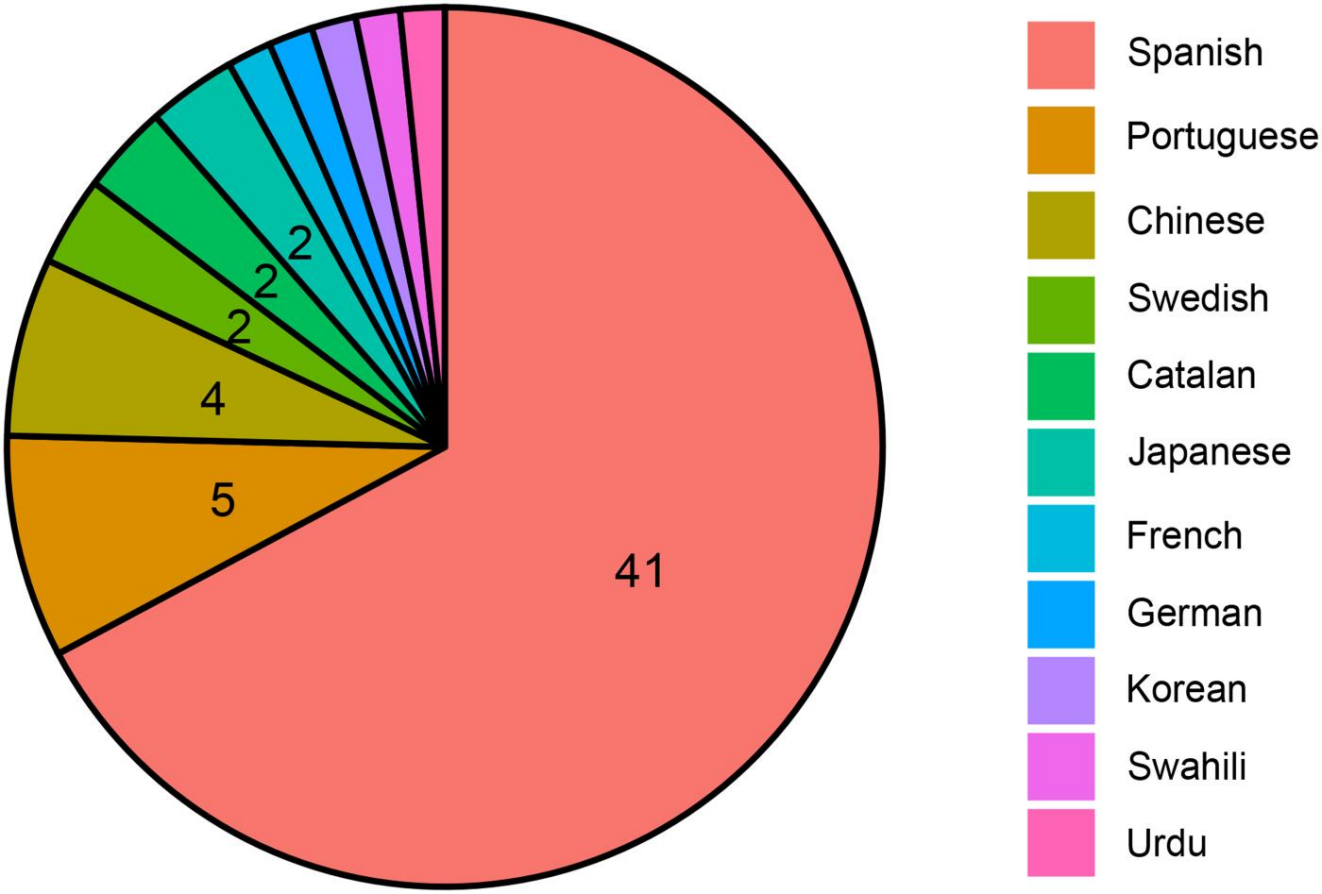
Pie chart showing the numbers of non-English competition entries, by language. Numbers are shown for languages with more than one entry.

Recent committee initiatives have focused on expanding and improving computational biology coverage in Spanish Wikipedia. Consequently, the majority of non-English competition entries have been in Spanish. However, several other languages have also seen multiple entries to the competition (Fig. 2).

A recurring motivational factor for entering the competition has been either the low quality, or lack of Wikipedia articles for computational biology learners. Vivek Rai won third place in the 2015 Wikipedia track for his contributions to the “ViennaRNA package” article (https://en.wikipedia.org/wiki/ViennaRNA_Package) and won first place in the 2015 Wikidata track for creating the corresponding Wikidata item (https://www.wikidata.org/wiki/Q21933168). Vivek commented:

At the time, I was studying RNA secondary structures for one of my university courses and was using the ViennaRNA software package for analysis. I was surprised to find that Wikipedia didn’t have an article on it yet, so it immediately struck me as the perfect candidate for my competition entry.

Yadi Zhou won first prize in 2016 for contributions to “Smith-Waterman algorithm” in English (https://en.wikipedia.org/wiki/Smith-Waterman_algorithm) and creating the corresponding article in Chinese Wikipedia. Yadi noted:

I chose this article as it was related to my research, but at the time it was not very informative; I felt I could make some significant improvements as part of my competition entry.

James Estevez was the winner of the first competition for his contributions to the “Genomics” article (https://en.wikipedia.org/wiki/Genomics). James recalled:

I had been fairly active on Wikipedia before the competition started. I was primarily interested in computational biology and bioinformatics and had been working on using my class notes to help improve the quality of those topics … the competition offered the opportunity to focus intensely on a specific article.

From the start, the competition was open to both individual entrants and teams. The power of collaborative editing was highlighted in the second competition, where the entries of two student teams placed in the top three articles. Team entries have since regularly placed in the top three, winning the competition on seven occasions since. While somewhat anecdotal, the observation that collaborative editing produces better text fits with established pedagogical research, as we have discussed previously (Sélem-Mojica *et al.* 2025); the individual benefits of collaborative writing and editing for students have been suggested to extend beyond the group exercise (Lopres *et al.* 2023), indicating a lasting benefit of entering the competition.

As in previous publications, we emphasise the unique educational benefits of the competition for individual participants as well. These include: (a) refining their research skills by identifying reliable sources that meet Wikipedia’s guidelines (prioritising secondary sources over original research, as an example), (b) synthesizing their findings into accessible and usable text, and (c) practicing writing complex ideas for a lay audience on an open platform. One recurring comment from past participants was that this process solidified their knowledge of their chosen subject. Felix Richter, who won first prize for his contributions to the “RNA-Seq” article (https://en.wikipedia.org/wiki/RNA-Seq) in 2019, reflected:

It led me to review fundamental literature on the topic, and cemented and organised my knowledge of that topic.

Nicole Wheeler, who won third prize for her contributions to the “Pfam” article (https://en.wikipedia.org/wiki/Pfam) in 2016, added:

The competition definitely forced me to understand (the topic) at a level of depth that allowed me to summarise and explain it to a broad audience.

James Estevez viewed the competition as:

(A)nother example of that Feynman quote about not really knowing something until you try to explain it to someone else.

Vivek Rai concurred:

It forced me to think deeply about how to take a complex scientific topic and structure it logically for a future reader: essentially, to build the exact resource I had been looking for myself. It was a practical lesson in clear, accurate, and effective scientific communication.

## Future perspectives

As we look to the future, we consider the continued role of the ISCB Wikipedia Competition in enhancing an educational resource for a field that is starting to mature. While some articles will naturally stabilise, others will require ongoing updates, and some are yet to be created.

Many of the previous winners who shared their experiences with us for this article responded that they continued to edit Wikipedia after the competition, indicating a lasting impact beyond the competition’s editing period. Vivek Rai reflected:

My editing became more sporadic for a few years while I was focused on my PhD, but I never stepped away completely. Since finishing my doctorate, I’ve returned with a renewed dedication: it feels wonderful to be an active part of the community again.

Tiago Lubiana won second prize in 2019, 2020 and 2021 for his contributions to the “Multiomics,” “OBO Foundry,” and “Biocuration” articles (https://en.wikipedia.org/wiki/Multiomics; https://en.wikipedia.org/wiki/OBO_Foundry; https://en.wikipedia.org/wiki/Biocuration). Tiago commented:

I engaged more and more with the platforms after entering the competition. The competition also helped me in finding other people that were excited about the interface of Wikipedia and bioinformatics. In academia, we often work in niches that get little attention and little direct connection with people from “the outside.” On Wikipedia, we get to share knowledge more widely, and that’s very fulfilling.

Recent years have seen strengthened collaborations between the Wikipedia Committee and regional groups and conferences in Latin America, which have mainly focused on identifying and realising the potential for growth in Wikipedia articles relating to computational biology, written in native languages for that region. Our recent study quantified the “knowledge gap” in computational biology content between English and Spanish Wikipedias and illustrated the significant improvements in quality that can be made with a modest, yet focused, editing event (Sélem-Mojica *et al.* 2024). These collaborations have boosted numbers of non-English competition entries, highlighting the value of expanding similar outreach initiatives in other regions. Future efforts can be guided by the size and activity levels of the ISCB Regional Student Groups (Eranti *et al.* 2023), and by building on collaborations such as the SCS2021 hackathon (Kilpatrick *et al.* 2022).

We must also consider the role of artificial intelligence (AI) and large language models (LLMs) as potential tools for article creation. These are undoubtedly powerful technologies, and while machine translation is indeed already used to some extent in some Wikipedia projects (Alshahrani *et al.* 2023), as of mid 2025 the Wikipedia community consensus is to prefer human decisions over machine-generated outcomes until the implications of AI are better understood (https://en.wikipedia.org/wiki/WP:AI). While the 2025 edition of the ISCB Wikipedia Competition has no explicit rules regarding the use of AI and LLMs, and no entrants have declared using LLMs, we expect to include a statement for future editions mirroring the ISCB’s policy on acceptable uses of LLMs (https://www.iscb.org/iscb-policy-statements/iscb-policy-for-acceptable-use-of-large-language-models). While AI and LLMs can generate content, being a good editor and understanding the specifics of Wikipedia editing is a vital prerequisite to knowing how AI can help generate better content. Alex commented:

These are new challenges for the Wikipedia community, and for the competition. I see the competition as a training exercise: knowing the differences between scientific writing and writing Wikipedia articles, understanding the community and, of course, the science. So if you ask ChatGPT to write your article, it’s like asking ChatGPT to do your homework for you: you’ll get something at the end of the day, but it’s not going to help you much.

A recent study suggested that we may also see a reduced reliance on Wikipedia in the wake of LLMs and web search summaries generated by AI tools (Wagner and Jiang 2025). The full impact of LLMs on Wikipedia use remains to be seen, but studies have already indicated that Wikipedia editing and viewing activity declines when articles resemble LLM-generated content, which could eventually affect the quality of future training data and future of human-curated knowledge (Lyu *et al.* 2025). Wikipedia remains one of the frontiers where human oversight and involvement still plays a central role in knowledge creation, refinement, and dissemination. Further, in a field such as computational biology, where methods evolve rapidly and accurate communication is critical, maintaining reliable, accessible, and globally relevant content is crucial. To this end, initiatives such as ISCB Student Wikipedia competitions play a key role and serve as models for other fields in demonstrating how formal communities of practice can accomplish these goals.

Finally, we leave potential competition entrants with some advice from previous awardees. Yadi Zhou:

Make every edit count, and only focus on improving a specific section or problem at one time.

Anton Pashkov and Sofía González-Sánchez (joint third prize in 2023 for their contributions to the “DNA annotation” article (https://en.wikipedia.org/wiki/DNA_annotation)):

Don’t make huge changes all at once. Uploading tiny and gradual modifications helps keep track of your progress and makes it easier for other editors to review them.

Nicole Wheeler:

Have a go! It’s a great chance to get research and writing experience, and (Wikipedia) is a great community to be a part of.

Tiago Lubiana:

Edit a bit every day, get acquainted with the details of Wikipedia. The world is watching, but you are capable enough, so be bold. And have fun!
